# The psychometric properties and applicability of subjective cognitive measures used in menopause research: a systematic review

**DOI:** 10.1097/GME.0000000000002494

**Published:** 2025-03-24

**Authors:** Lexi He, Nicole G. Jaff, Emily Kontaris, Aimee Spector

**Affiliations:** From the 1 Division of Psychology and Language Sciences, Department of Clinical, Educational and Health Psychology, University College London, United Kingdom; 2 Department of Chemical Pathology, National Health Laboratory Service, Faculty of Health Sciences, University of the Witwatersrand, Johannesburg, South Africa; 3 School of Psychology, Faculty of Health and Medical Sciences, University of Surrey, Guildford, United Kingdom.

**Keywords:** Cognition, Menopause, Psychometric properties, Subjective cognitive complaints

## Abstract

**Importance:**

Many midlife women report cognitive issues when they transition through menopause. These cognitive complaints affect women's mental health and quality of life. However, the current understanding of women's cognitive experiences during the menopause transition has been limited by the lack of validated self-reported cognitive measures.

**Objective:**

This systematic review aimed to identify existing self-reported, or subjective, cognitive measures used in menopause research and evaluate their psychometric properties and applicability.

**Evidence Review:**

Three databases, Medline, Embase, and PsycINFO, were searched in March 2024 with no restriction on publication year. Studies investigating women transitioning into postmenopause and with cognitive experiences measured using validated subjective cognitive measures were selected. The assessment of psychometric properties and applicability of included measures was conducted based on their development process and their performance in the menopause studies selected.

**Finding:**

Twenty-eight menopause studies involving 15 measures were included. Included measures showed adequate content validity, internal consistency, and construct validity when they were developed, yet other psychometric properties were either poor or not reported. Hence, the overall performance of included measures was generally moderate to poor. Information relating to psychometric properties of included measures in menopause studies was also lacking, indicating doubtful applicability.

**Conclusions and Relevance:**

Poor psychometric properties or the lack of psychometric assessment of existing subjective cognitive measures may indicate doubt or uncertainty regarding their applicability in women transitioning through menopause. This review recommends the use of subjective cognitive measures that assess more than one cognitive domain, as well as further assessment of the psychometric properties of these measures before their use in menopause research or clinical settings, particularly those measures initially developed for clinical practice. It also highlights the need for future development of a subjective cognitive measure for women transitioning through menopause to improve the current understanding of their cognitive challenges.

## Abstract 2

This systematic review found that the psychometric properties and applicability of existing subjective cognitive measures used with women transitioning into postmenopause was doubtful and recommends future development of a subjective cognitive measure for menopause research and clinical practice.

**Key points**

• **Objective:** This study aimed to investigate the psychometric properties and applicability of subjective cognitive measures used in menopause research.

• **Findings:** Twenty-eight studies involving 15 measures were included. Included measures performed moderately to poorly in the assessment of psychometric properties. The applicability of included measures with women during the menopause transition was doubtful.

• **Meaning:** This systematic review identifies a need to develop a subjective cognitive measure specifically designed for people during the menopause transition with satisfactory psychometric properties.

With the increasing life expectancy, people including women, transgender, and nonbinary people (both the terms “women” and “people” are used to acknowledge that some nonbinary or transgender people also experience menopause due to changes in hormonal states)^1,2^ spend several years transitioning into postmenopause. This period when people transition from the reproductive to the postreproductive phase is known as the menopause transition (MT).^3^ The fluctuation in the hormonal milieu during this time has been found to contribute to symptoms experienced during this period. Common menopausal symptoms include hot flashes, mood changes, sleep disturbance, and brain fog. The ensuing menopausal symptoms are intricately related, causing an additional effect on women's menopausal experiences.^4^

Self-reported, or subjective cognitive issues, including difficulty concentrating and poor memory, are among the most prevalent menopausal complaints reported by over 60% of women.^5^ These complaints have been validated in longitudinal studies measuring objective cognitive performance, which found a minor yet significant decline in cognitive functioning in perimenopausal women as compared to pre- and postmenopausal women.^6^ In addition, women's cognitive complaints have consistently been found to be correlated with neuropsychological test performance of attention and working memory, and less consistently with verbal memory.^6^ Despite the high prevalence of cognitive complaints and significant decline in objective cognition at MT, only around 11% to 13% of people may develop cognitive impairment.^7^

Nevertheless, many neuropsychological tests used to measure objective cognition were designed to help diagnose cognitive disorders, such as the Mini-Mental State Examination^8^ for people with dementia. These tests may lack the sensitivity and ecological validity to capture the subtle changes experienced during the MT.^7^ This highlights the importance of subjective cognitive measures when women are not cognitively impaired but still report a decline in cognitive functioning during the MT.

Furthermore, the correlation between subjective cognitive experience and mental well-being during the MT has been consistently found.^9-11^ It has been suggested that there might be a psychological component in self-reported menopausal symptoms^12^ and the possibility of interventions to enhance menopausal experiences by improving cognitive experiences. This again highlights the importance of measuring people's cognitive experiences in clinical practice to help understand their overall menopausal experiences.

Our current understanding of women's subjective cognitive experiences at MT has been limited due to the lack of validated subjective cognitive measures. To our knowledge, only one measure was developed specifically for women at MT — the 20-item Cognitive Symptom Index for Midlife Women.^13,14^ It consists of eight items reflecting cognitive changes (eg, forgetfulness, primary items) and 12 items of other menopausal symptoms purported to be strongly related to (eg, depression, secondary items) or might be related to cognitive symptoms (eg, hot flashes, tertiary items). However, some primary items (eg, worrying) might not directly reflect cognitive changes, and the total cognitive symptoms are calculated by summing up all three types of items despite the differences in the strength of correlation between cognitive changes and item types. A considerable number of studies measured cognitive experiences with the Menopause Rating Scale,^15^ which contains one cognition-related item — physical and mental exhaustion. However, this item does not specify the cognitive difficulties women experience, such as the types of memory lapses.^5,16^ Other studies implemented questionnaires developed for other populations, such as people with dementia.^17^ To our knowledge, no studies have systematically identified these measures and assessed their psychometric properties and applicability in the context of menopause.

The purpose of this systematic review is to (1) identify existing subjective cognitive measures that have been used for women at MT, (2) critically appraise the psychometric properties of these measures, and (3) evaluate the applicability of these measures in menopause research. We aim to provide recommendations regarding the instruments used to measure women's cognitive experiences during the MT.

## METHODS

This systematic review has been preregistered on PROSPERO (CRD42023477642) and followed the PRISMA 2020 guideline.^18^

### Search strategy

Searches were conducted on three databases, Medline, Embase, and PsycINFO, via the UCL Ovid interface from March 10 to March 15, 2024 with no restrictions of publication year. Search terms were the combinations of three key concepts: “menopause” AND “cognition” AND “subjective measures.” Search terms for menopause included the following: “Menopause” OR “Perimenopause” OR “Premenopause” OR “Postmenopause” OR “Midlife women” OR “Climacter*” OR “Menopause transition” OR “Middle aged women” OR “Ovariectomy” OR “Oophorectomy” OR “Salpingo-oophorectomy.” Search terms for cognition included the following: “Attention” OR “Alertness” OR “Brain fog” OR “Cognition” OR “Cognitive impairment” OR “Cognitive function” OR “Cognitive challenges” OR “Cognitive decline” OR “Cognitive dysfunction” OR “Concentration” OR “Forget” OR “Executive function” OR “Learning” OR “Memory” OR “Metamemory” OR “Verbal fluency” OR “Processing speed” OR “Visuospatial” OR “Motor” OR “Recall” OR “Psychomotor.” Search terms for subjective measures included the following: “Subjective” OR “Experience” OR “Self” OR “Self-experienced” OR “Self-reported” OR “Self-rating” OR “Self-evaluation” OR “Self-perceived” OR “Perception” OR “Patient-reported” OR “Complaint” OR “Concern” OR “Survey” OR “Questionnaire” OR “Scale” OR “Quiz” OR “Measure” OR “Test” OR “Instrument” OR “Subscale” OR “Tool” OR “Battery.” These search terms and the MeSH terms were adapted for each database. Truncations were used where appropriate. The reference lists of included studies were reviewed for additional studies.

### Study selection

All records found were exported to Rayyan (https://www.rayyan.ai), which is an online software for study selection. Duplicates were detected automatically by Rayyan and removed manually. A title and abstract screening were performed, followed by a full-text screening. Studies that the first reviewer (L.H.) found ambiguous were screened independently by the second reviewer (E.K.) and discussed between the two reviewers (L.H. and E.K.) until decisions were made for inclusion in the review. Authors of inaccessible papers were contacted twice, and papers with no replies would be excluded.

The inclusion criteria were papers:

1. focusing on people transitioning through any type of menopause and who were staged at the pre-, peri-, and/or postmenopausal stage or with surgical or treatment-induced menopause, which cannot be staged;
2. using validated outcome measure(s) of subjective cognition with the above participants;
3. published in peer-reviewed journals.

The exclusion criteria were papers:

1. written in non-English and translations unavailable;
2. not focused on menopause;
3. inaccessible;
4. with only subitems of subjective cognition.

### Data extraction

After full-text screening, the subjective cognitive measures were identified from the included studies. The development papers of identified measures were retrieved for the assessment of psychometric properties. For measures with more than one development paper, all were retrieved.

Data regarding the psychometric properties of included measures (eg, measurement aim, target population, construct being measured, and psychometric analyses) were extracted from the development papers to a preprepared Excel worksheet to critically appraise the psychometric properties of the subjective cognitive measures identified. Data regarding the study designs, participants' characteristics, and findings relating to psychometric properties of the subjective cognitive measures were extracted from the included menopause studies on another preprepared Excel worksheet to narratively assess the applicability of the above measures in menopause research.

### Quality assessment

The Quality Criteria for Measurement Properties by Terwee and colleagues^19^ were used to assess the psychometric properties of included measures when they were developed. The Terwee criteria assess eight domains of psychometric properties regarding the development process of a measure. These domains are content validity, internal consistency, criterion validity, construct validity, reproducibility (ie, agreement and reliability), responsiveness, floor and ceiling effect, and interpretability. Definitions of these eight domains and rating instructions can be found in Table 1. For the convenience of scoring, “2”, “1,” or “0” points were granted if the criteria were fully met (“+” in Table 1), partially met (“?” in Table 1), or not met or no information (“−/0” in Table 1), respectively, as in previous review.^20^ The total score can range from 0 to 18. A total score that falls into the first quartile of the total score (0-4) was considered “poor” in quality, the second (5-9) was considered “moderate” in quality, the third (10-13) was considered “good” in quality, and the fourth (14-18) was considered “excellent” in quality. Ratings were combined for measures with more than one development paper. Two reviewers (L.H. and E.K.) conducted the quality assessment independently. A consensus meeting was held to discuss disagreements until consensus was reached.

**TABLE 1:** Terwee criteria for the quality assessment*^a^*

Property	Definition	Quality criteria*^b,c^*	
1. Content validity	The extent to which the domain of interest is comprehensively sampled by the items in the questionnaire	+	A clear description is provided of the measurement aim, the target population, the concepts that are being measured, and the item selection AND target population and (investigators OR experts) were involved in item selection
		?	A clear description of above-mentioned aspects is lacking OR only target population involved OR doubtful design or method
		–	No target population involvement
		0	No information found on target population involvement
2. Internal consistency	The extent to which items in a (sub)scale are intercorrelated, thus measuring the same construct	+	Factor analyses performed on adequate sample size (7 × no. items and ≥100) AND Cronbach's *alpha*(s) calculated per dimension AND Cronbach's *αlpha*(s) between 0.70 and 0.95
		?	No factor analysis OR doubtful design or method
		–	Cronbach's *αlpha*(s) <0.70 or >0.95, despite adequate design and method
		0	No information found on internal consistency
3. Criterion validity	The extent to which scores on a particular questionnaire relate to a gold standard	+	Convincing arguments that gold standard is “gold” AND correlation with gold standard ≥0.70
		?	No convincing arguments that gold standard is “gold” OR doubtful design or method
		–	Correlation with gold standard <0.70, despite adequate design and method
		0	No information found on criterion validity
4. Construct validity	The extent to which scores on a particular questionnaire relate to other measures in a manner that is consistent with theoretically derived hypotheses concerning the concepts that are being measured	+	Specific hypotheses were formulated AND at least 75% of the results are in accordance with these hypotheses
		?	Doubtful design or method (eg, no hypotheses)
		–	<75% of hypotheses were confirmed, despite adequate design and methods
		0	No information found on construct validity
5. Reproducibility			
5.1 Agreement	The extent to which the scores on repeated measures are close to each other (absolute measurement error)	+	MIC < SDC OR MIC outside the LOA OR convincing arguments that agreement is acceptable
		?	Doubtful design or method OR (MIC not defined AND no convincing arguments that agreement is acceptable)
		–	MIC ≥ SDC OR MIC equals or inside LOA, despite adequate design and method
		0	No information found on agreement
5.2 Reliability	The extent to which patients can be distinguished from each other, despite measurement errors (relative measurement error)	+	ICC or weighted *κ* ≥ 0.70
		?	Doubtful design or method (eg, time interval not mentioned)
		–	ICC or weighted *κ* < 0.70, despite adequate design and method
		0	No information found on reliability
6. Responsiveness	The ability of a questionnaire to detect clinically important changes over time	+	SDC or SDC < MIC OR MIC outside the LOA OR RR >1.96 OR AUC ≥0.70
		?	Doubtful design or method
		–	SDC or SDC ≥ MIC OR MIC equals or inside LOA OR RR ≤1.96 OR AUC <0.70, despite adequate design and methods
		0	No information found on responsiveness
7. Floor and ceiling effects	The number of respondents who achieved the lowest or highest possible score	+	≤15% of the respondents achieved the highest or lowest possible scores
		?	Doubtful design or method
		–	>15% of the respondents achieved the highest or lowest possible scores, despite adequate design and methods
		0	No information found on interpretation
8. Interpretability	The degree to which one can assign qualitative meaning to quantitative scores	+	Mean and SD scores presented of at least four relevant subgroups of patients and MIC defined
		?	Doubtful design or method OR less than four subgroups OR no MIC defined
		0	No information found on interpretation

AUC, receiver operating characteristics curve; ICC, intraclass correlation; LOA, limits of agreement; MIC, minimum important change; RR, Guyatt's responsiveness ratio; SD, standard deviation; SDC, smallest detectable change.

*^a^*Table reprinted from Journal of Clinical Epidemiology Vol 60/1, Terwee CB, Bot SDM, de Boer MR, et al. Quality criteria were proposed for measurement properties of health status questionnaires, 34-42, Copyright (2006), with permission from Elsevier.^19^

^*b*^“+” = 2 points; “?” = 1 point; “–” = 0 point; “0” = 0 point.

*^c^*Doubtful design or method = no description of study designs or methods, poor study designs or methods, or insufficient sample sizes (N < 50).

## RESULTS

**FIG. 1:**
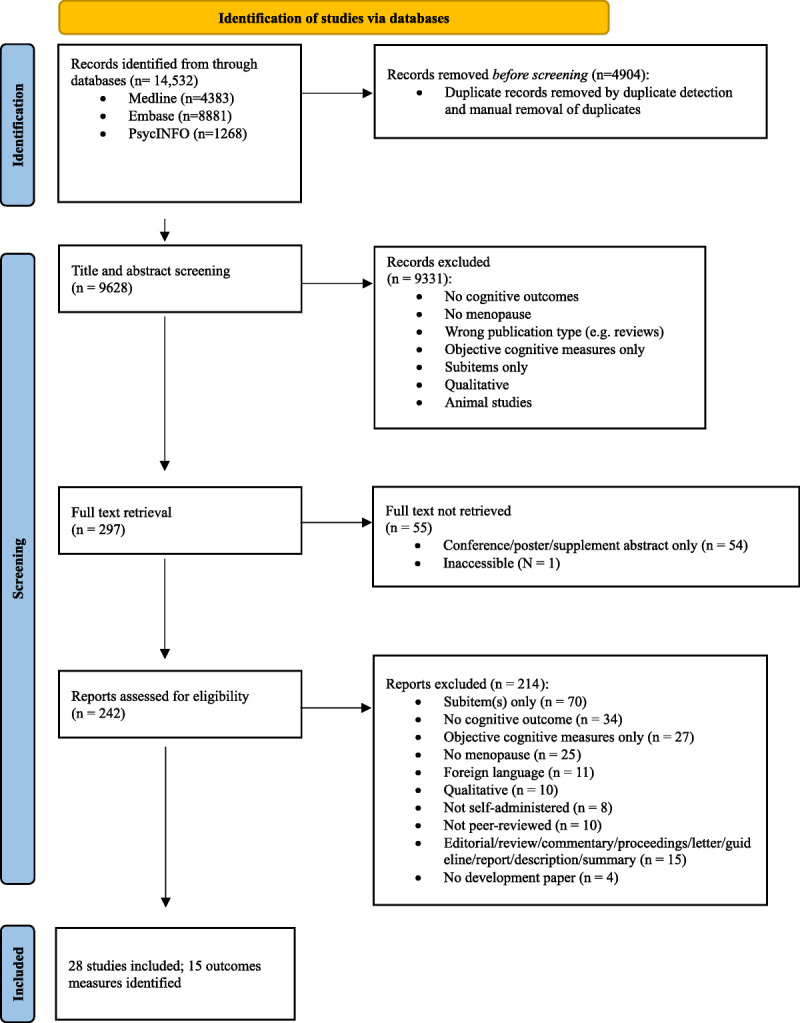
Flow diagram of study selection.

Figure 1 outlines the flow of study selection. After the removal of duplicates, 9,628 studies were screened for titles and abstracts. After the title and abstract screening, 9,331 studies were excluded leaving 297 studies. Among these, 55 studies were either inaccessible or did not have full texts and thus were removed. Of the remaining 242 studies, 214 studies were excluded. The most common reason was the absence of a validated subjective cognitive measure (N = 70). The 20-item Cognitive Symptom Index for Midlife Women^13,14^ developed for women during the MT was excluded due to a lack of development paper. A total of 28 studies were included, identifying 15 subjective cognitive measures.

### Characteristics of included studies and measures

Included studies measured the subjective cognition of premenopausal, perimenopausal, postmenopausal, surgical postmenopausal, and/or treatment-induced postmenopausal women using validated subjective cognitive measures with randomized controlled trials (RCTs; N = 7), longitudinal (N = 5), and cross-sectional designs (N = 16). Twenty-one studies included women with natural and/or induced menopause, and seven did not specify menopause types.

The fifteen measures identified from the above studies were categorized by the cognitive domains they aim to measure when developed (Table 2). The majority (N = 10) measured one cognitive domain (ie, either attention or memory), and five measured multiple cognitive domains. Seven out of 15 measures were developed for clinical samples and the remaining for nonclinical samples.

**TABLE 2:** Characteristics of included measures

Construct	Measures	No. of items, No. of domains	Target population Domain(s) measured
Attention	AFI^21^	13 Items, 3 domains	Breast cancer patients 1. Effective action 2. Attentional lapses 3. Interpersonal effectiveness
	BADDS^22,23^	40 Items, 5 domains	Adolescences and adults 1. Organizing and activating to work 2. Sustaining attention and concentration 3. Sustaining energy and effort 4. Managing affective interference 5. Utilizing working memory and accessing recall
Memory	EMQ,^24^ EMQ-R^25^	EMQ: 35 items, 5 domains;EMQ-R: 13 items, 3 domains	EMQ: people with severe closed head injury 1. Speech 2. Reading and writing 3. Faces and places 4. Actions 5. Learning new things EMQ-R: neurological patients 1. Memory retrieval 2. Attentional tracking 3. Two items share little in common: forgetting places of things and forgetting what has been read before
	MemCO^26^	12 Items, 4 domains	Adults 1. Present memory ability 2. Potential improvement in memory ability 3. Effort utility 4. Inevitable decrement in memory
	MFQ^27–29^	64 Items, 4 domains	Adults and older adults 1. Frequency of forgetting 2. Retrospective functioning 3. Seriousness of forgetting 4. Mnemonic usage
	MIA^30–32^	120 Items, 8 domains	Adults 1. Knowledge of memory strategies 2. Knowledge of memory tasks and processes 3. Knowledge of own memory capacities 4. Attitudes toward own memory 5. Activities supportive of memory 6. Memory and state anxiety 7. Memory and achievement motivation 8. Locus of control in memory abilities
	MMQ^33^	61 Items, 3 domains	Older adults 1. Memory contentment 2. Memory ability 3. Memory strategy
	PRMQ^34,35^	16 Items, 8 domains	People with Alzheimer's disease and their carers 1. Eight domains from the eight combinations among self-cued or environmental cued, long-term or short-term memory, retrospective or prospective memory
	SMCQ^36^	14 Items, 2 domains	Older adults 1. General memory 2. Everyday memory
	SMQ^37,38^	43 Items, 1 domain, and 36 items	Healthy population and patients with temporal lobectomy 1. Organization of behavior 2. The factor loadings of the remaining 36 items were too small to be a dimension
Multi cognitive domains	CCI^39^	125 Items, a combination of six questionnaires	Older adults with cognitive complaints 1. Six questionnaires of self-rating or informant-rating memory functioning
	CFQ^40^	25 Items, 3 domains	Adults 1. Perception 2. Memory 3. Motor function
	FACT-Cog,^41^ FACT-Cog v3^42^	37 Items, 4 domains (FACT-Cog v3)	Oncology patient 1. Perceived cognitive impairment 2. Perceived cognitive ability 3. Noticeability (from others) 4. Quality of life
	MACCS^43^	28 Items, 4 domains	People with OCD and community sampled adults without OCD 1. Beliefs about general memory abilities 2. Confidence in decision-making abilities 3. Confidence in one's ability to focus or concentrate 4. High standards about one's cognitive performance
	MOS-cog^44^	6 Items, 6 domains	Patients in clinical settings 1. Memory 2. Attention 3. Reasoning 4. Judgement 5. Reaction time 6. Confusion

AFI, Attentional Function Index; BADDS, Brown Attention Deficit Disorder Scale; CCI, Cognitive Complaint Index; CFQ, Cognitive Failure Questionnaire; EMQ, Everyday Memory Questionnaire; EMQ-R, Everyday Memory Questionnaire–Revised; FACT-Cog, Functional Assessment of Cancer Therapy-Cognitive; FACT-Cog v3, FACT-Cog version 3; MACCS, Memory and Cognitive Confidence Scale; MemCO, Memory Controllability Inventory; MFQ, Memory Functioning Questionnaire; MIA, Metamemory in Adulthood; MMQ, Multifactorial Memory Questionnaire; MOS-cog, Medical Outcomes Study Cognitive Functioning Scale; OCD, obsessive-compulsive disorder; PRMQ, Prospective and Retrospective Memory Questionnaire; SMCQ, Subjective Memory Complaints Questionnaire; SMQ, Subjective Memory Questionnaire.

### Assessment of psychometric properties

The development papers of included measures were subject to a quality assessment based on the Terwee criteria. The summary of quality ratings after reviewers' consensus meeting is shown in Table 3.

**TABLE 3:** Ratings of the psychometric properties for included measures

	Terwee criteria*^a^*									
Outcome measures	Content validity	Internal consistency	Criterion validity	Construct validity	Agreement	Reliability	Responsiveness	Floor/ceiling effect	Interpretability	Total
AFI	+	+	0	+	0	0	0	0	0	
	2	2	0	2	0	0	0	0	0	6
BADDS	+	?	+	+	0	?	?	0	+	
	2	1	2	2	0	1	1	0	2	11
CCI	?	0	0	+	0	0	0	0	?	
	1	0	0	2	0	0	0	0	1	4
CFQ	?	?	0	?	0	−	0	0	0	
	1	1	0	1	0	0	0	0	0	3
EMQ	+	0	0	?	0	0	0	0	?	
	2	0	0	1	0	0	0	0	1	4
EMQ/EMQ-R	+	+	0	?	0	0	0	0	?	
	2	2	0	1	0	0	0	0	1	6
FACT-Cog/FACT-Cog version 3	+	?	0	0	0	0	0	−	0	
	2	1	0	0	0	0	0	0	0	3
MACCS	?	+	0	+	0	?	0	0	?	
	1	2	0	2	0	1	0	0	1	7
MemCo	+	−	0	+	0	?	0	0	0	
	2	0	0	2	0	1	0	0	0	5
MFQ	+	+	0	+	0	0	0	0	?	
	2	2	0	2	0	0	0	0	1	7
MIA	+	−	0	+	0	0	0	0	?	
	2	0	0	2	0	0	0	0	1	5
MMQ	+	?	0	+	0	?	0	0	?	
	2	1	0	2	0	1	0	0	1	7
MOS-cog	?	?	0	+	0	0	0	0	0	
	1	1	0	2	0	0	0	0	0	4
PRMQ	+	+	0	0	0	0	0	−	?	
	2	2	0	0	0	0	0	0	1	5
SMCQ	+	+	+	+	0	?	+	0	?	
	2	2	2	2	0	1	2	0	1	12
SMQ	+	?	0	−	0	?	0	0	?	
	2	1	0	0	0	1	0	0	1	5

AFI, Attentional Functional Index; BADDS, Brown Attention Deficit Disorder Scale; CCI, Cognitive Complaint Index; CFQ, Cognitive Failure Questionnaire; EMQ, Everyday Memory Questionnaire; EMQ/EMQ-R, Everyday Memory Questionnaire/Revised; FACT-Cog/FACT-Cog version 3, Functional Assessment of Cancer Therapy-Cognitive/ Functional Assessment of Cancer Therapy-Cognitive version 3; MACCS, Memory and Cognitive Confidence Scale; MemCo, Memory Controllability Inventory; MFQ, Memory Functioning Questionnaire; MIA, Metamemory in Adulthood; MMQ, Multifactorial Memory Questionnaire; MOS-cog, Medical Outcomes Study Cognitive Functioning Scale; PRMQ, Prospective and Retrospective Memory Questionnaire; SMCQ, Subjective Memory Complaints Questionnaire; SMQ; Subjective Memory Questionnaire.

*^a^*There were two rows for each measure. The first row contains the ratings according to the Terwee criteria showed on Table 1, and the second row contains the numerical scores assigned to each rating. The plus sign “+” equals to “2” points; the question mark “?” equals to “1” point; the minus sign “−” and the zero sign “0” equals to 0 points.

#### Attention

Two scales measure attention-related functioning: the 13-item Attentional Function Index (AFI)^21^ and the 40-item Brown Attention Deficit Disorder Scale (BADDS).^22,23^ Although the AFI has been used since 1992,^45^ its development process was reported in 2011,^21^ and this 2011 paper is thus included in this review as the development paper of the AFI. These scales earned moderate and good scores, respectively (6/18 for AFI; 11/18 for BADDS).

Both measures showed high construct validity with different constructs (ie, other subjective cognitive measures, mood measures, and neuropsychological tests of attention) and high content validity. The AFI showed good internal consistency evidenced with a factor model as theoretically expected and acceptable Cronbach's alpha (*α* = 0.80-0.92), whereas the internal consistency of the BADDS was undetermined due to the absence of a factor analysis. A significant difference was found in the BADDS scores between clinical and nonclinical samples as categorized by the gold standard, the Diagnostic and Statistical Manual of Mental Disorders III^46^ of attention deficit disorders (ADD), indicating criterion validity (*P* < 0.0001).^22^ Cutoff points differentiating clinical and nonclinical samples provided interpretations for the BADDS scores. Longitudinal data provided evidence for BADDS's test-retest reliability and responsiveness. No other psychometric properties were reported for the BADDS and AFI.

#### Memory

Eight measures assess memory-related functioning. These are the Everyday Memory Questionnaire/Revised (EMQ/EMQ-R),^24,25^ Memory Controllability Inventory (MemCo),^26^ Memory Functioning Questionnaire (MFQ),^27-29^ Metamemory in Adulthood,^30-32^ Multifactorial Memory Questionnaire (MMQ),^33^ Prospective and Retrospective Memory Questionnaire (PRMQ),^34,35^ Subjective Memory Complaints Questionnaire (SMCQ),^36^ and Subjective Memory Questionnaire (SMQ).^37,38^ Both the ratings for EMQ and ratings combining EMQ and EMQ-R were reported as two studies used the EMQ^47^ and the EMQ-R,^48^ respectively.

All memory functioning measures performed moderately in psychometric properties, except for the EMQ (4/18) with poor scores and the SMCQ (12/18) with good scores. All showed good content validity and internal consistency except for the MemCo and Metamemory in Adulthood, which showed Cronbach's alphas less than 0.7. The significant correlations between memory measures and objective cognition, mood, and other subjective cognitive measures indicated high construct validity,^24,26,29,31,33,36^ except for the PRMQ with no information reported and SMQ with no significant correlations with related constructs. Four longitudinal development papers (ie, MemCo, MMQ, SMCQ, and SMQ)^26,33,36,37^ showed unspecified test-retest reliability either because they did not specify the methods of computing test-retest reliability or because they had insufficient sample sizes (N < 50). Floor and ceiling effects were found in the PRMQ.^34^ Although floor and ceiling effects were found in three items of the EMQ,^24^ whether these effects were present in EMQ total scores was not reported. The SMCQ, which aimed to help detect dementia, showed good criterion validity.^36^ Other psychometric properties of memory measures were generally neglected in scale development.

#### Multidomain measures

Five measures assessed self-reported cognitive functioning in a variety of domains. These are the Cognitive Complaint Index (CCI),^39^ Cognitive Failure Questionnaire (CFQ),^40^ Functional Assessment of Cancer Therapy—Cognitive (FACT-Cog)^41^ and its version three (FACT-Cog version 3),^42^ Memory and Cognitive Confidence Scale (MACCS),^43^ and Medical Outcomes Study Cognitive Functioning Scale (MOS-cog).^44^

The psychometric properties of multidomain measures were generally poor (≤4/18 in CCI, CFQ, FACT-Cog, and MOS-cog; 7/18 in MACCS). All measures showed evidence of content validity and internal consistency. Correlations between these measures (except for the FACT-Cog and FACT-Cog version 3) and brain volumes, obsessive-compulsive disorder symptoms, psychological well-being, mood, and other subjective cognitive measures were found as hypothesized, showing construct validity.^39,40,43,44^ Both MACCS (*r* = 0.74-0.94)^43^ and CFQ (*r* = 0.86)^40^ showed test-retest reliability, although the developers of the CFQ did not expect this test-retest reliability, as the CFQ was developed to measure a temporary state of cognitive functioning reflecting current work stress. A ceiling effect was found in the FACT-Cog. Although the CCI^39^ and MACCS^43^ development papers reported and compared the means and standard deviations of subgroups, a minimal important change for interpretation was not defined, hence, their interpretability was unclear. Information regarding criterion validity, reproducibility, floor and ceiling effect, and responsiveness was commonly lacking.

### Applicability of included measures

The applicability of included measures was interpreted narratively. Although women complained about multiple cognitive domains in previous studies,^5^ most existing measures focused on only one cognitive domain (ie, attention or memory) as shown in Table 2, highlighting a problem of their content validity. Furthermore, none of the measures were developed for women at MT as shown in Table 2, and none of the studies except for Zhu and colleagues^48^ specifically aimed to assess the psychometric properties of included measures with women at MT. However, other studies^9-11,47-71^ incidentally provided evidence for the psychometric properties of included measures among their main findings. The performance of included measures in their development studies and their performance when used in menopause research was compared. The characteristics and psychometric-related findings of included measures are in Table 4.

**TABLE 4:** Characteristics and the psychometric-related findings of included studies

Measure (Terwee total)	Author(s)	Study description	Participants (methods of menopausal staging)
	Psychometric properties	
Sample size	Menopause paper	Development paper*^a^*
AFI (7)	Grummisch et al, 2023^10^	Longitudinal study investigating the impact of within-person hormonal changes on cognition	Perimenopausal women aged 45-55 years (STRAW+10)	N = 43	1. Construct validity: – AFI and Perceived Stress Scale Score (*r* = −0.63, *P* < 0.01) – AFI and the CES-D (*r* = −0.74, *P* < 0.01)	1. Construct validity: – AFI and POMS-SF confusion subscale (*r* = −0.59, *P* < 0.01) – AFI and SDS concentration item (*r* = −0.58, *P* < 0.01) – AFI and CFQ (*r* = −0.60, *P* < 0.01)
BADDS (11)	Epperson et al, 2011^49^	An RCT investigating the treatment effect of atomoxetine on menopause-related cognitive decline	Perimenopausal and postmenopausal women aged 45-60 years (perimenopause: last menstrual period <12 months with irregular menstrual cycle for at least 6 months, a cycle duration of ≤21 or ≥ 35 days, and a serum FSH level > 20 IU/L; postmenopausal: last menstrual period >12 months with FSH level > 35 IU/L)	N = 16	1. Responsiveness: – Treatment effect of atomoxetine on BADDS scores (num *df* = 1, ATS = 6.8, *P* = 0.009)	1. Construct validity: – BADDS scores and the attention-related components in the WISC-III and WAIS-R 2. Responsiveness: – Treatment effect of a stimulant medication for treating attention deficit disorder on the decline in BADDS scores
	Epperson et al, 2015^50^	An RCT investigating the treatment effect of LDX on cognitive function in women with midlife-onset executive function difficulties	Perimenopausal and early postmenopausal women aged 45-60 years with midlife-onset executive function difficulties (perimenopause: irregular menstrual cycle for ≥12 months, no menstrual period for ≥3 months and a serum FSH level ≥20 IU/L; postmenopause: no period for ≥12 months, within 5 years of their last menstrual period, and FSH ≥35 IU/L)	N = 32	1. Responsiveness: – Treatment effect of LDX on the BADDS scores (num *df* = 1, ATS = 15.42, *P* < 0.001)	
	Page et al, 2023^51^	Cross-sectional study investigating the impact of menopause on executive functioning	Pre-, perimenopausal, natural, or surgical postmenopausal women aged 35-65 years (STRAW for natural menopause)	N = 1,971	1. Construct validity: – The BADDS scores and menopausal status (at least *P* < 0.001) after adjusted for age, education, ADHD, sleep difficulty, anxiety, and depression	
	Shanmugan et al, 2017^52^	An RCT investigating the treatment effect of LDX in midlife women with midlife-onset executive function difficulties	Perimenopausal and early postmenopausal women aged 45-60 years with BADDS scores ≥20 during the MT and postmenopause (staging as Epperson et al,^50^ 2015 above)	N = 14	1. Responsiveness: – Treatment effect of LDX on BADDS total and subscales scores (*F* = 26.6, *df* = 13, *P* < 0.001) – Correlations between LDX-induced changes in BADDS scores and in right insula activation (*r* = 0.95, *P* < 0.001)	
	Shanmugan et al, 2020^53^	Cross-sectional study investigating the relationship among adverse childhood experience, mood symptoms, and cognition	Women aged 30-73 years who had undergone RRSO (no staging)	N = 552	1. Construct validity: – BADDS scores and Adverse Childhood Experience scores (aMD = 7.1, *P* < 0.001)	
CCI (4)	Conley et al, 2020^54^	Cross-sectional brain imaging study investigating the association between subjective cognitive complaints and brain cortical structure	Postmenopausal women aged 50-60 years with natural menopause (STRAW+10)	N = 44	1. Construct validity: – CCI and the gray-matter volume in the right medial temporal area (*r* = −0.445, *P* < 0.002, *R*^2^ = 0.2) – CCI and MSC (*β* = 36.62, *P* < 0.002) – CCI and BDI (*β* = 10.2, *P* = 0.006)	1. Construct validity: – Higher CCI and the reduced gray-matter density in distributed brain regions (*P* < 0.001)
	Conley et al, 2022^55^	An RCT investigating the effect of E2 treatment on subjective cognitive complaint	Postmenopausal women aged 50-60 with natural menopause (STRAW+10)	N = 40	1. Construct validity: – CCI and 0-back blocks (*r* = −0.41, *P* = 0.01) and 1-back blocks (*r* = −0.34, *P* = 0.036) of the N-back working memory task	
	Dumas et al, 2013^56^	Cross-sectional study investigating the brain activation in postmenopausal women with or without cognitive complaints	Postmenopausal women aged 50-60 years with nonsurgical menopause (nonsurgically induced amenorrhea for 1 year and beyond)	N = 23	1. Construct validity: – CCI and BDI-II (*t*_21_ = 2.50, *P* = 0.02) – CCI and differences in brain activation in the middle frontal gyrus (*P* < 0.005 for all *t* tests)	
	Vega et al, 2016^57^	Cross-sectional brain imaging study investigating the brain connectivity in postmenopausal women with or without cognitive complaints	Postmenopausal women aged 50-60 years with nonsurgical menopause (staging as in Dumas et al,^56^ 2013 above)	N = 31	1. Construct validity: – CCI and MSC (*r* = 0.69, *P* < 0.01) – CCI and BDI (*r* = 0.49, *P* < 0.01) – CCI and brain connectivity between dorsolateral frontal cortex and right middle frontal gyrus (*r* = 0.73, *P* < 0.01), and right fusiform gyrus (*r* = 0.65, *P* < 0.01)	
	Vega et al, 2018^58^	Cross-sectional study comparing cancer-related and menopause-related cognitive complaints	Group 1: Breast cancer, ovarian cancer, or lymphoma cancer patients aged 35-80 years who underwent chemotherapy (no staging) Group 2: postmenopausal women aged 50-60 years with nonsurgical menopause (staging as in Dumas et al,^56^ 2013 above)	N = 63	1. Construct validity: – CCI in oncology patients and healthy postmenopausal controls (*F*_2,60_ = 70.73, *P* < 0.001) – CCI and MSC (*r* = 0.60, *P* < 0.001, *R*^2^ = 0.357) – CCI and BDI (*r* = 0.50, *P* < 0.001, *R*^2^ = 0.252) – CCI and the CRT task (*r* = 0.36, *P* = 0.005, *R*^2^ = 0.126)	
CFQ (3)	Jenkins et al, 2006^59^	Longitudinal studies investigating the effects of adjuvant treatments on cognition in women with breast cancer	Women with early breast cancer, with chemotherapy or nonchemotherapy, with or without treatment-induced menopause, and pre-, peri-, and postmenopausal healthy controls (staging not specified)	N = 177	1. Responsiveness: – Treatment effect of chemotherapy on CFQ scores four weeks after the therapy (*t* = −4.24, *P* < 0.001) – Treatment effect of nonchemotherapy treatment on CFQ scores 12 months after therapy (*F* = 13.34, *P* = 0.001)	1. Responsiveness: no information
EMQ (4)	Ford et al, 2004^47^	Cross-sectional questionnaire study investigating women's subjective memory at MT	Women aged 25-64 years in pre-, peri-, postmenopausal, HT, or hormonal contraceptive group (premenopausal: a regular menstrual pattern; perimenopausal: an irregular menstrual pattern in the past 12 months; postmenopausal: amenorrhea for 12 months and beyond; no staging for the HT and hormone contraceptive groups)	N = 172	1. Internal consistency: – Cronbach’s *α* = 0.91 2. Construct validity: – EMQ and HADS (*F* = 74.81, *df* = 1, *P* < 0.001) – EMQ scores predicted from the WHQ and the HADS (*F* = 28.57, *df* = 1,125, *P* < 0.001, *R*^2^ = 0.30) 3. Floor and ceiling effect: – More than 15% of participants (15.84%-22.57%) scored maximum of the EMQ total scores	1. Internal consistency: no information 2. Construct validity: – Participants' and informants' EMQ total scores in patient groups (*r* = 0.45-0.58, *P* < 0.01) 3. Floor and ceiling effect: – Zero was the modal score for 3 items out of 29 EMQ items
EMQ-R (6)	Zhu et al, 2023^48^	A study evaluating the psychometric properties of EMQ-R with women at MT	Women aged 40-60 years at pre-, peri- or early postmenopausal stage (STRAW+10)	N = 417	1. Internal consistency: – A confirmatory factor analysis conducted yielded a two-factor model as hypothesized – Cronbach’s *α* = 0.77-0.88 2. Construct validity: – EMQ-R retrieval subscale and menopausal status (*F* = 3.17, *P* = 0.04)	1. Internal consistency: – A principal component analysis yielded three factors as theoretically hypothesized – Cronbach’s *α* = 0.89 2. Construct validity: no information
FACT-Cog/FACT-Cog version 3 (3)	Chang et al, 2020^60^	Longitudinal study investigating the impact of surgical induced menopause on cognition	Premenopausal women aged 30-54 years who had undergone RRSO (no staging)	N = 57	1. Responsiveness: – The impact of oophorectomy on the decreases in the Perceived Cognitive Impairment subscale of FACT-Cog at 6-month and 12-month postsurgery (*P* < 0.001)	1. Responsiveness: no information
MACCS (7)	Ballantyne et al, 2021^61^	Intervention study investigating the effects of cognitive remediation intervention on women at MT	Perimenopausal, natural, or surgical postmenopausal women aged 40-65 years (staging not specified)	N = 27	1. Responsiveness: – An intervention effect on the MACCS scores at immediate postintervention with moderate to large effect sizes (*b* = −2.90, SE = 0.62, *t_26_* = −4.69, *P* < 0.001, *d* = 0.90)	1. Responsiveness: no information
MemCo (5)	Unkenstein et al, 2016^62^	Cross-sectional study investigating women's subjective memory complaints at MT	Pre-, peri- and postmenopausal women aged 40-60 years (STRAW+10)	N = 130	1. Internal consistency: – Cronbach’s *α* = 0.84 2. Construct validity: – MemCo and MMQ-frequency of forgetting subscale (*r* = 0.37, *P* = 0.007)	1. Internal consistency: – A confirmatory factor analysis conducted yielded four factors as theoretically expected – Cronbach’s *α* = 0.58-0.70 for Present Ability; 0.62-0.75 for Potential Improvement; 0.65-0.73 for Effort Utility; and 0.58-0.77 for Inevitable Decrement 2. Construct validity: – MemCo subscales and Ageing Concern Questionnaire (*r* = −0.54 to 0.052, *P* < 0.05) – MemCo subscales and the Rosenbaum Self-Control Schedule (*r* = −0.33 to 0.24, *P* < 0.05) – MemCo and the Personality in Intellectual Contexts Scale (*r* = −0.38 to 0.57, *P* < 0.01)
MFQ (7)	Drogos et al, 2013^9^	Cross-sectional study investigating the relationship between objective and subjective cognition in women with vasomotor symptoms at MT	Women aged 44-62 years with at least 35 hot flashes per week (with last menstruation within 6 months to 10 years)	N = 68	1. Construct validity: – MFQ-Current memory subscale and the PANAS — negative affect (*β* = −0.25, SE = 0.02, *P* < 0.05), and CVLT (*β* = 0.23, SE = 0.13, *P* < 0.05) – MFQ-Frequency of forgetting subscale and PANAS — negative affect (*β* = −0.22, SE = 0.02, *P* < 0.05), and DSF (*β* = −0.25, SE = 0.05, *P* < 0.05) – MFQ-Retrospective functioning subscale and PANAS-positive affect (*β* = 0.34, SE = 0.01, *P* < 0.001) and the Greene Climacteric Scale-vasomotor subscale (*β* = 0.23, SE = 0.07, *P* < 0.05)	1. Internal consistency: – An exploratory factor analysis and confirmatory factor analysis were conducted – Cronbach’s *α* = 0.83-0.94 2. Construct validity: – MFQ-Frequency of forgetting subscale and immediate list recall (*β* = 0.32, *t* = 3.93, *P* < 0.01), delayed list recall (*β* = 0.27, *t* = 3.16, *P* < 0.01), and recognition (*β* = 0.26, *t* = 3.10, *P* < 0.01). – MFQ-Seriousness of forgetting and list recognition (*β* = −0.17, *t* = −2.19, *P* < 0.05) – MFQ-Frequency of forgetting subscale scores predicted the Randt Memory test of acquisition (*β* = 0.31, *t* = 2.24, *P* < 0.05) and delayed recall (*β* = 0.33, *t* = 2.44, *P* < 0.05) 3. Responsiveness: no information
	Grummisch et al, 2023^10^	As above	As above	As above	1. Construct validity: – MFQ and the Perceived Stress Scale (*r* = −0.43, *P* < 0.05) – MFQ and the PSQI (*r* = −0.18, *P* < 0.01) – MFQ and the CES-D (r = −0.53, *P* < 0.01) – MFQ and vasomotor symptom numbers (*r* = −0.09, *P* < 0.05), and vasomotor scores (*r* = −0.13, *P* < 0.01) – MFQ and estrone glucuronide levels (*r* = −0.15, *P* < 0.01)	
	Maki et al, 2007^63^	An RCT investigating the impact of hormone therapy on cognition and quality of life	Postmenopausal women aged 45-55 years (last menstruation period ≥12 months and ≤36 months)	N = 180	1. Responsiveness: – Treatment effect of hormone therapy in MFQ-seriousness of forgetting subscale (mean change, 4.96; *P* = 0.028)	
	Weber and Mapstone, 2009^64^	Cross-sectional study investigated the relationship between subjective and objective cognition in perimenopausal women	Perimenopausal women aged 40-60 years (STRAW)	N = 24	1. Construct validity: – MFQ and the RAVLT encoding (*r* = 0.48, *P* = 0.02) and LNS (*r* = −0.44, *P* = 0.03). – MFQ and the BDI (*r* = −0.71, *P* < 0.001), WHQ somatic subscale (*r* = 0.50, *P* = 0.013), and WHQ sleep subscale (*r* = 0.46, *P* = 0.024)	
	Weber et al, 2012^11^	Cross-sectional study investigating the relationship between subjective cognition and objective cognition at MT	Perimenopausal women aged 40-60 years (STRAW)	N = 75	1. Construct validity: – MFQ and LNS (*r* = 0.28, *P* = 0.015) – MFQ and D2 Test of Attention (*r* = 0.26, *P* < 0.05) – MFQ and WHQ-somatic subscale (*r* = 0.449, *P* < 0.001) – MFQ and WHQ-sleep subscale (*r* = 0.312, *P* = 0.006) – MFQ and BDI (*r* = −0.529, *P* < 0.001) – MFQ and BAI (*r* = −0.334, *P* = 0.003)	
	Woods et al, 2000^65^	Cross-sectional study investigating women's subjective cognition at MT	Midlife women aged 55-85 years in the early/middle/late perimenopausal, postmenopausal or hormone use group (early perimenopausal: no changes in flow or cycle length since age 35 years; middle perimenopausal: irregular cycles without skipping periods since age 35 years; late perimenopausal: one or more skipping periods since age 35 years; postmenopausal: amenorrhea for 12 months or beyond without any other reasons for amenorrhea; HT group: no staging)	N = 205	1. Internal consistency: – Cronbach’s *α* = 0.84-0.95 2. Construct validity: – MFQ subscales and CES-D (*r* = 0.16-0.32, *P* < 0.05) – MFQ subscales and Perceived Stress (*r* = 0.19-0.22, *P* < 0.05) – MFQ subscales and Perceived Health Status (*r* = −0.39 to −0.19, *P* < 0.01) – MFQ-Current memory subscale scores predicted from the menopausal status (*F_4,167_* = 3.95, *P* = 0.004)	
	Schneider et al, 2019^66^	RCTs investigating the safety and feasibility of a formulation of phytoestrogen, on menopausal symptoms	Perimenopausal and postmenopausal women aged 45-60 years (last natural menstrual cycle ≥60 days and <4 years)	N = 71	No psychometric outcomes regarding the MFQ	
Metamemory in Adulthood (5)	Unkenstein et al, 2016^62^	As above	As above	As above.	1. Internal consistency (only MIA Achievement subscale): – Cronbach’s *α* = 0.80 2. Construct validity: – MIA-achievement subscale and MMQ-memory contentment (*r* = −0.43, *P* = 0.002)	1. Internal consistency: – Cronbach’s *α* = 0.28-0.76 for the Activity domain 2. Construct validity: – MIA total and text memory performance (*r* = 0.23-0.47, *P* < 0.05)
MMQ (7)	Unkenstein et al, 2016^62^	As above	As above	As above.	1. Internal consistency: – Cronbach’s *α* = 0.89-0.96 2. Construct validity: – MMQ and menopausal status (*F_10,246_* = 2.46, *P* = 0.008, *η*^2^ = 0.09) – MMQ-frequency of forgetting subscale and MemCo total (*r* = 0.37, *P* = 0.007) – MMQ-contentment subscale and MemCo total (*r* = 0.42, *P* = 0.002), and MIA-achievement subscale (*r* = −0.43, *P* = 0.002) – MMQ Ability and DSF (*r* = −0.27, *P* = 0.046) – MMQ Contentment and MAS (*r* = 0.48, *P* = 0.011)	1. Internal consistency: – A principal component analysis was conducted – Cronbach’s *α* = 0.83-0.95 2. Construct validity: – MMQ subscales and MIA subscales (*r* = −0.57 to 0.61, *P* < 0.001) – MMQ subscales and MFQ subscales (*r* = 0.45-0.70, *P* = 0.001) 3. Responsiveness: no information
	Unkenstein et al, 2017^67^	Intervention study investigating the efficacy of a memory strategy program	Perimenopausal women aged 45-60 years (STRAW+10)	N = 32	1. Internal consistency: – Cronbach’s *α* = 0.81-0.92 at all time points 2. Responsiveness: – Intervention effect found in all three MMQ subscales (all *P* < 0.05)	
MOS-cog (4)	Terra et al, 2023^68^	Online questionnaire study investigating the impact of premenopausal and postmenopausal RRSO on cognition	Women with premenopausal RRSO aged ≤45 years and women with postmenopausal RRSO aged ≥54 years(no staging)	N = 641	1. Construct validity: – MOS-cog and depression diagnosis (OR, 1.7-3.1) – MOS-cog reasoning subitem and menopausal status at RRSO (OR, 1.8; 95% CI, 1.1-3.1) – MOS-cog multitasking subitem and menopausal status at RRSO (OR, 1.9; 95% CI, 1.1-3.4)	1. Construct validity: – Other sets of the Medical Outcomes Study questionnaires on mental well-being (all *P* < 0.001)
PRMQ (5)	Piauilino et al, 2010^69^	Population-based survey study investigating prospective and retrospective memory	People aged 20-80 years (STRAW)	N = 322	1. Internal consistency – A confirmatory factor analysis found a factor model as theoretically expected – Cronbach’s *α* = 0.77-0.89 2. Construct validity: – PRMQ and menopausal status with moderate effect sizes (*F*_1,319_ = 3.75, MSE = 2.26, *P* < 0.03)	1. Internal consistency: – A confirmatory factor analysis conducted with eight factors as theoretically expected – Cronbach’s *α* = 0.80-0.89 2. Construct validity: no information
SMCQ (11)	Kim et al, 2023^70^	Cross-sectional study investigating psychosomatic symptom clusters in Korean perimenopausal women	Korean perimenopausal women aged 40 years or more (STRAW)	N = 1,060	1. Internal consistency: Cronbach’s *α* = 0.83 2. Construct validity: – The latent analysis conducted indicated some evidence for the correlations between the SMCQ and menopausal symptoms (ie, the Overactive Bladder Symptom Score, Female Sexual Function Index-5, Insomnia Severity Index, Patient Health Questionnaire-9, Patient Health Questionnaire-15, and the Somatic Symptom Severity Scale)	1. Internal consistency: – A confirmatory factor analysis was conducted – Cronbach’s *α* = 0.69-0.83 2. Construct validity: – All three SMCQ subscales and the Camdex Memory Complaint Questionnaire, Seoul Informant Report Questionnaire for Dementia, Geriatric Depression Scale, and clinical cognitive function and neuropsychological tests (all *P* < 0.01)
SMQ (5)	Gorenstein et al, 2011^71^	An RCT investigating the impact of estrogen therapy on verbal memory in postmenopausal women	Hysterectomized midlife postmenopausal women aged 40-59 years (FSH level >40 mUI/mL)	N = 53	1. Floor and ceiling effect: – No evidence for floor or ceiling effect as the SMQ scores in placebo and treatment group were both within the medium range	1. Floor/ceiling effect: no information

ADHD, Attention Deficit hyperactivity Disorder; AFI, attentional function index; aMD, adjusted mean difference; ATS, ANOVA-type statistic; BADDS, brown attention deficit disorder scale; BAI, Beck's Anxiety Inventory; BDI, Beck's Depression Inventory; CCI, cognitive complaint index; CES-D, Center for Epidemiological Studies Depression scale; CFQ, cognitive failure questionnaire; CRT, choice reaction time; CVLT, California Verbal Learning Test; DAS, Distributed Antenna System ;DSF, Digit Span Forward; E2, estradiol; EMQ, everyday memory questionnaire; EMQ-R, everyday memory questionnaire/revised; FACT-Cog, functional assessment of cancer therapy—cognitive; FSH, follicle-stimulating hormone; HADS, Hospital Anxiety and Depression Scale; HT, hormone therapy; LDX, lisdexamfetamine; LNS, Letter-Number Sequencing; MT. menopause transition; MAS, Menopause Attitude Scale; MSC, Menopause Symptom Checklist; num *df*, numerator degrees of freedom; MSE, mental status examination; MACCS, Memory and Cognitive Confidence Scale ; MemCO, Memory Controllability Inventory; MFQ, Memory Functioning Questionnaire; MIA, Metamemory in Adulthood; MMQ, Multifactorial Memory Questionnaire; MOS-cog, Medical Outcomes Study Cognitive Functioning Scale; PANAS, Positive and Negative Affect Schedule; POMS-SF. Profile of Mood States; PRMQ, Prospective and Retrospective Memory Questionnaire; PSQI, Pittsburgh Sleep Quality Index; RCT, randomized controlled trials; RAVLT, Rey Auditory Verbal Learning Test; RRSO, risk-reducing salpingooophorectomy; SDC, smallest detectable change; SMCQ, Subjective Memory Complaints Questionnaire; SMQ, SubjectiveMemory Questionnaire; SE, standard error; STRAW, 2001 Staging of Reproductive Aging Workshop^72^; STRAW+10, 2011 Staging of Reproductive Aging Workshop^3^; WAIS-R, Wechsler Adult Intelligence Scale—Revised; WHQ, Women's Health Questionnaire; WISC-III, Wechsler Intelligence Scale for Children III.

*^a^*Some development papers also assess other psychometric properties of measures; only the psychometric properties corresponding to what was found in the menopause studies were presented here for the purpose of comparison.

Included menopause studies provided evidence for four psychometric properties domains in the Terwee criteria — construct validity (N = 11 measures), internal consistency (N = 7 measures), responsiveness (N = 6 measures), and floor and ceiling effect (N = 2 measures), and other psychometric properties were not reported.

Included measures performed similarly in internal consistency and construct validity when they were used in menopause research as compared to when they were developed. Specifically, all memory measures (except for the SMQ) showed adequate Cronbach's *α*s as detailed in the Terwee criteria (ie, 0.70 ≥ α ≤0.95).^47,48,62,65,67,69,70^ Significant correlations between included measures and related constructs (eg, depressive symptoms) indicated their construct validity,^9-11,47,48,51,53-58,62,64,65,68-70^ except for the FACT-Cog, MACCS, and SMQ with no information regarding construct validity. Depressive symptom measures^10,11,47,54,56-58,64,65,68,70^ and neuropsychological tests of attention and working memory^9,11,55,62,64^ were consistently found to be correlated with different subjective cognition measures. The correlation between subjective cognition and objective verbal memory was less consistent, and all significant correlations were found to be with the MFQ.^9,64^

However, included intervention studies and RCTs provided evidence regarding measures' responsiveness and floor and ceiling effect, which were not found in their development papers. Specifically, evidence supported the responsiveness of the MMQ, MACCS, FACT-Cog, and BADDS in detecting changes in pre- and posttreatment subjective cognition.^49,50,52,59-61,63,67^ Studies also found that a ceiling effect of EMQ^47^ in women during the MT limited the applicability of this measure. Information regarding the other four psychometric properties was absent in included studies.

## DISCUSSION

To our knowledge, this is the first systematic review to evaluate the psychometric properties and applicability of subjective cognitive measures used in menopause research. This systematic review found that existing measures had good content validity, internal consistency, and construct validity when they were developed and most also showed good construct validity when they were used with women transitioning through menopause. Specifically, the correlations between subjective cognition and objective attention, working memory, and verbal memory were found across studies, especially when the subjective cognition was measured with the MFQ, validating people's cognitive complaints. The correlation between subjective cognition and depressive symptoms was constantly found in women at MT across studies, suggesting potential for menopause intervention to improve psychological well-being by improving subjective cognition. Other psychometric properties were either poor or not reported, giving rise to uncertainty about the applicability of existing subjective cognitive measures.

Among all the psychometric criteria being assessed in this review, the lack of assessment regarding the floor and ceiling effect and content validity when measures were used in menopause research may be detrimental to an understanding of women's menopausal and cognitive experiences. This is because nearly half the measures were developed for clinical samples. However, it is widely recognized that women's cognitive changes during the MT can be subtle and transient, and the majority of women will not develop cognitive difficulties.^7^ These measures may not be adequately sensitive to effectively capture the cognitive changes. For example, a ceiling effect was found in the EMQ when it was used in perimenopausal women,^47^ which was developed for people with head injuries.^24^ This was also found in another measure developed for clinical samples described in this review.^34^ It is thus recommended that the seven measures developed for clinical samples should be adapted and validated before their use with women in menopause research.

Two-thirds of the included measures in this review mainly focus on either attention or memory functioning. Although women did complain most about memory and attention in previous studies,^7^ a possible explanation is that most measures focused on these two cognitive domains. Women's complaints in other cognitive domains (eg, slow thinking and reasoning)^68^ measured with the multidomain measures found in this review also supported this argument. Similarly, previous qualitative studies also found women to report nonmemory and nonattention cognitive complaints.^16^ Therefore, this review recommends future research to use scales that measure multiple cognitive domains.

However, findings from this review suggested that existing multidomain cognitive measures lacked psychometric properties. Furthermore, three of the five measures (ie, CFQ, FACT-Cog version 3, MOS-cog) have only been used in women with nonnatural menopause (based on the studies which specified menopause types).^59,60,68^ Additionally, these measures varied substantially in scale sizes. For example, the CCI with 114 items might be fatiguing for women who are experiencing brain fog, and MOS-cog with six items measuring six domains and EMQ-R with 13 items might be inadequate to comprehensively capture women's cognitive experiences.^48^ There is therefore a need for the development of a new subjective cognitive measure, which includes relevant cognitive domains within a suitable scale size.

### Strength and limitations

One strength of this systematic review is that it inclusively identified and appraised the applicability of existing subjective cognitive measures used in menopause research regardless of the different menopause types and methods of menopausal staging. This provides a comprehensive review and assessment of existing measures used in menopause research. However, findings relating to menopausal stage and cognition should be considered with caution, as some included studies may not be accurately staged with STRAW+10 — they are still included in this review with the population of interest. Furthermore, RCTs and longitudinal studies might have an advantage over cross-sectional studies with the Terwee criteria, as they allow comparisons across time points and thus might provide evidence for responsiveness, which is impossible for cross-sectional designs. Moreover, the criterion validity in the Terwee criteria might be less relevant to subjective cognitive measures, as there is currently no gold standard defined in this field. Nonetheless, this validity was still considered a criterion in this review, as two included measures (ie, the BADDS and SMCQ) were developed to help with clinical diagnosis. Moreover, all included studies considered only “women.” Although women are the majority who experience menopause, future investigation should also explore the cognitive experiences of nonbinary and transgender people who may experience menopausal symptoms as a result of changes in dosage of gender-affirming hormone therapy or the need to cease it but are highly underrepresented in the literature.

## CONCLUSIONS

This systematic review identified and appraised the psychometric properties of fifteen existing subjective cognitive measures in the context of menopause. The included measures generally performed moderately to poorly in the quality assessment of their psychometric properties. The applicability of included measures for women transitioning into menopause was uncertain due to the lack of psychometric evaluation, the inadequacy of domains being measured, and scale sizes. Future investigation should recognize the strengths and limitations of existing measures and develop a subjective cognitive measure that is more related to women's cognitive experiences during the MT.
